# 4512 Allopregnanolone Dose Finding for Status Epilepticus Treatment by Pharmacokinetic-Pharmacodynamic Modeling using Quantitative EEG in Dogs

**DOI:** 10.1017/cts.2020.51

**Published:** 2020-07-29

**Authors:** Edward “Ned” Patterson, Irene Vuu, Dorota Zolkowska, Chun-Yi Wu, Ilo Leppik, Greg Worrell, Vaclav Kremen, James Cloyd

**Affiliations:** 1 University of Minnesota CTSI; 2 University of Minnesota College of Veterinary Medicine; 3 University of California Davis Medical School; 4 University of Minnesota College of Pharmacy; 5 Mayo Clinic

## Abstract

OBJECTIVES/GOALS: Allopregnanolone (ALLO), a modulator of GABA_A_ receptors, may be useful as a treatment for human and canine benzodiazepine-refractory status epilepticus (SE). Our objective was to develop a phamacokinetic-pharmacodynamic (PKPD) model relating ALLO plasma concentrations to electroencephalographic (EEG) effects in dogs. METHODS/STUDY POPULATION: Four healthy dogs and one dog with epilepsy that had implanted intracranial electrodes were utilized. ALLO doses ranging from 1-6 mg/kg were administered IV over 5 min. EEG data were collected during four IM doses (1-2 mg/kg). Blood samples were collected up to 6 hr following dosing. ALLO concentrations were measured using HPLC-MS/MS. Power density was determined in EEG bands using a custom algorithm. A two-compartment link PKPD model was developed to describe the relation between ALLO plasma concentration and change in EEG power in the alpha, beta, delta and theta bands. RESULTS/ANTICIPATED RESULTS: ALLO caused a rapid increase in absolute power density in all EEG bands measured (1-4, >4 – 8, >8 – 12, >12 – 25, and >25 – 100 Hz). The onset of effect was rapid (1-3 min) and demonstrated by frequency band and dose analysis. Concentration-EEG data were best fit by a two-compartment PK model and sigmoidal Emax PD indirect link model. The beta frequency band was most sensitive, showing increases in power at the lowest ALLO concentrations. The EC50 concentration for the beta frequency was ~270 ng/mL. The EC50 values for effects on the other frequency bands were ~500-700 ng /mL. In conclusion, IV ALLO causes a rapid effect on EEG that can be used to determine minimal plasma concentrations associated with target engagement. DISCUSSION/SIGNIFICANCE OF IMPACT: Dose selection for future clinical trials will use the effective concentrations determined here in conjunction with studies in animal status epilepticus models. Studies are planned in client owned dogs with epilepsy to evaluate clinical efficacy in dogs and as nonclinical proof-of-concept evidence supporting translational studies in people. CONFLICT OF INTEREST DESCRIPTION: Michael Rogawski and Dorota Zolkowska are named as inventors on patent applications claiming use of neuroactive steroids including allopregnanolone and ganaxolone in the treatment of status epilepticus.

